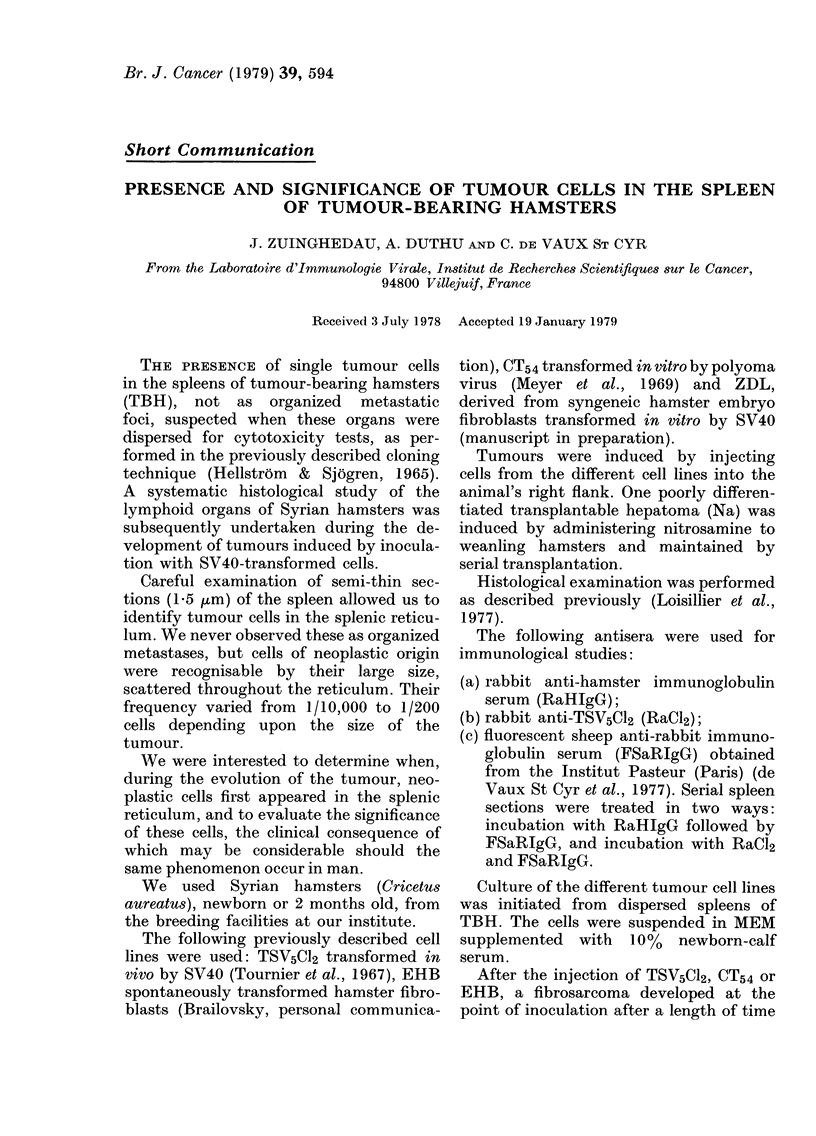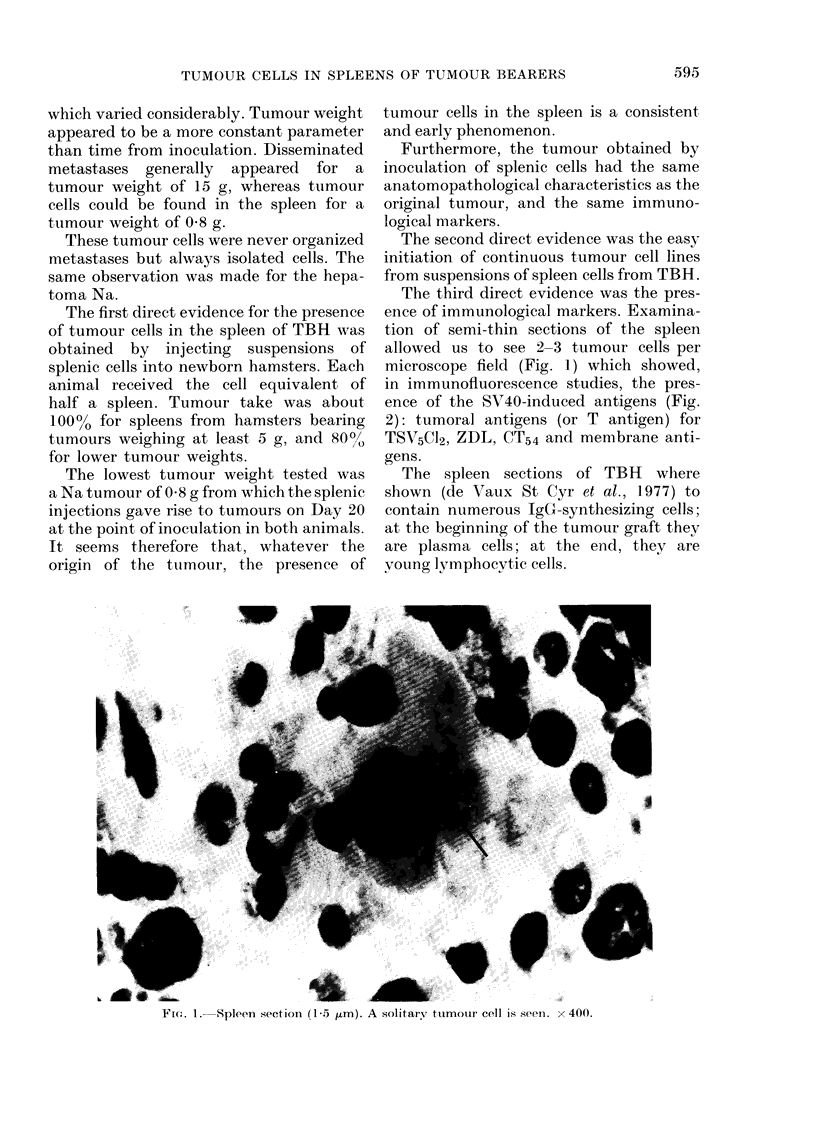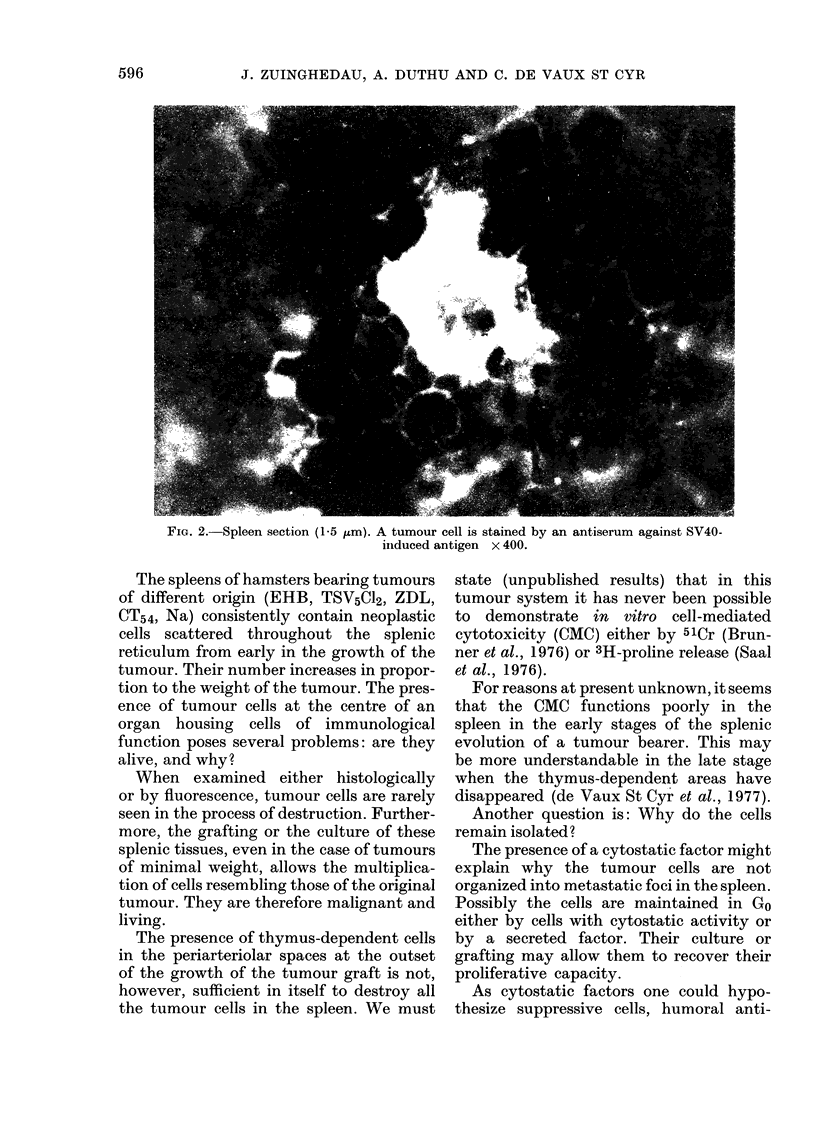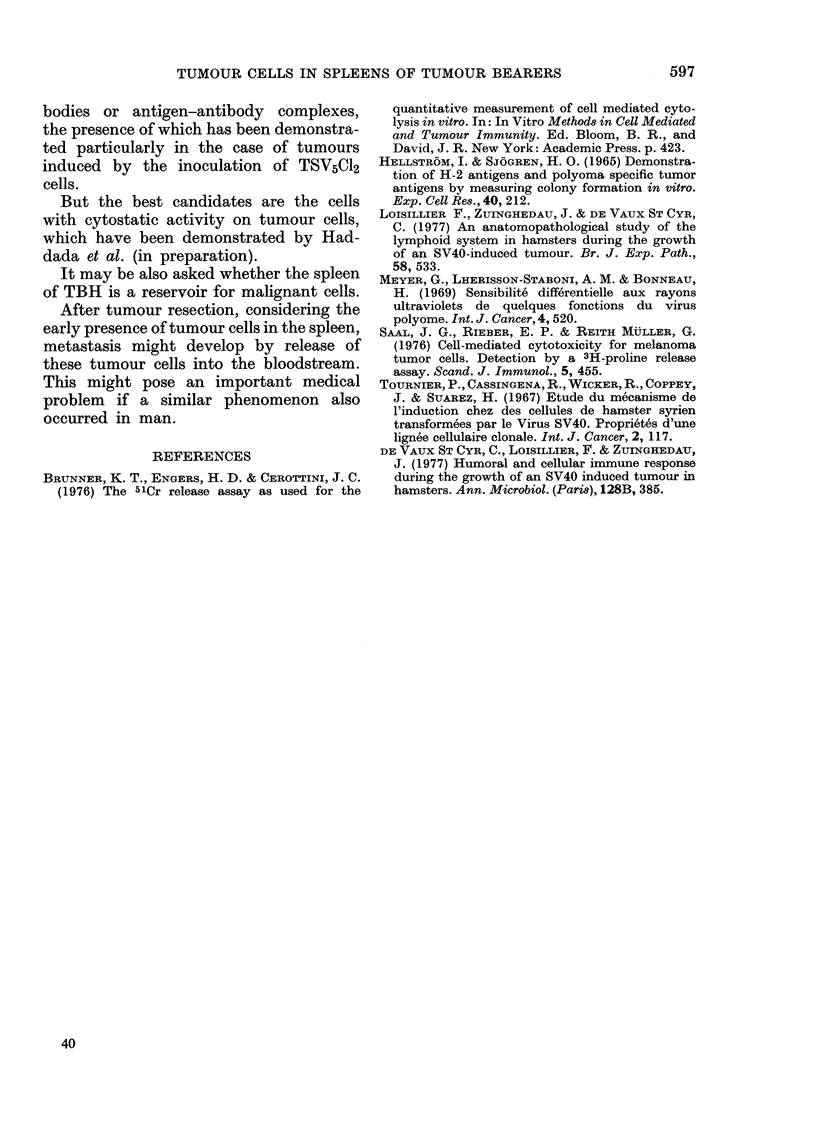# Presence and significance of tumour cells in the spleen of tumour-bearing hamsters.

**DOI:** 10.1038/bjc.1979.108

**Published:** 1979-05

**Authors:** J. Zuinghedau, A. Duthu, C. de Vaux St Cyr

## Abstract

**Images:**


*Br. J. Cancer* (1979) **39**, 594

***Short Communication***

### PRESENCE AND SIGNIFICANCE OF TUMOUR CELLS IN THE SPLEEN OF TUMOUR-BEARING HAMSTERS

J. ZUINGHEDAU, A. DUTHU AND C. DE VAUX ST CYR

*From the Laboratoire d'Immunologie Virale, Institut de Recherches Scientifiques sur le Cancer, 94800 Villejuif, France*

Received 3 July 1978 Accepted 19 January 1979

THE PRESENCE of single tumour cells in the spleens of tumour-bearing hamsters (TBH), not as organized metastatic foci, suspected when these organs were dispersed for cytotoxicity tests, as performed in the previously described cloning technique (Hellström & Sjögren, 1965). A systematic histological study of the lymphoid organs of Syrian hamsters was subsequently undertaken during the development of tumours induced by inoculation with SV40-transformed cells.

Careful examination of semi-thin sections (1·5 $\mu$m) of the spleen allowed us to identify tumour cells in the splenic reticulum. We never observed these as organized metastases, but cells of neoplastic origin were recognisable by their large size, scattered throughout the reticulum. Their frequency varied from 1/10,000 to 1/200 cells depending upon the size of the tumour.

We were interested to determine when, during the evolution of the tumour, neoplastic cells first appeared in the splenic reticulum, and to evaluate the significance of these cells, the clinical consequence of which may be considerable should the same phenomenon occur in man.

We used Syrian hamsters (*Cricetus aureatus*), newborn or 2 months old, from the breeding facilities at our institute.

The following previously described cell lines were used: $TSV_5Cl_2$ transformed *in vivo* by SV40 (Tournier *et al.*, 1967), EHB spontaneously transformed hamster fibroblasts (Brailovsky, personal communication), $CT_{54}$ transformed *in vitro* by polyoma virus (Meyer *et al.*, 1969) and ZDL, derived from syngeneic hamster embryo fibroblasts transformed *in vitro* by SV40 (manuscript in preparation).

Tumours were induced by injecting cells from the different cell lines into the animal's right flank. One poorly differentiated transplantable hepatoma (Na) was induced by administering nitrosamine to weanling hamsters and maintained by serial transplantation.

Histological examination was performed as described previously (Loisillier *et al.*, 1977).

The following antisera were used for immunological studies:

(a) rabbit anti-hamster immunoglobulin serum (RaHIgG);
(b) rabbit anti-$TSV_5Cl_2$ ($RaCl_2$);
(c) fluorescent sheep anti-rabbit immunoglobulin serum (FSaRIgG) obtained from the Institut Pasteur (Paris) (de Vaux St Cyr *et al.*, 1977). Serial spleen sections were treated in two ways: incubation with RaHIgG followed by FSaRIgG, and incubation with $RaCl_2$ and FSaRIgG.

Culture of the different tumour cell lines was initiated from dispersed spleens of TBH. The cells were suspended in MEM supplemented with 10% newborn-calf serum.

After the injection of $TSV_5Cl_2$, $CT_{54}$ or EHB, a fibrosarcoma developed at the point of inoculation after a length of time which varied considerably. Tumour weight appeared to be a more constant parameter than time from inoculation. Disseminated metastases generally appeared for a tumour weight of 15 g, whereas tumour cells could be found in the spleen for a tumour weight of 0·8 g.

These tumour cells were never organized metastases but always isolated cells. The same observation was made for the hepatoma Na.

The first direct evidence for the presence of tumour cells in the spleen of TBH was obtained by injecting suspensions of splenic cells into newborn hamsters. Each animal received the cell equivalent of half a spleen. Tumour take was about 100% for spleens from hamsters bearing tumours weighing at least 5 g, and 80% for lower tumour weights.

The lowest tumour weight tested was a Na tumour of 0·8 g from which the splenic injections gave rise to tumours on Day 20 at the point of inoculation in both animals. It seems therefore that, whatever the origin of the tumour, the presence of tumour cells in the spleen is a consistent and early phenomenon.

Furthermore, the tumour obtained by inoculation of splenic cells had the same anatomopathological characteristics as the original tumour, and the same immunological markers.

The second direct evidence was the easy initiation of continuous tumour cell lines from suspensions of spleen cells from TBH.

The third direct evidence was the presence of immunological markers. Examination of semi-thin sections of the spleen allowed us to see 2–3 tumour cells per microscope field (Fig. 1) which showed, in immunofluorescence studies, the presence of the SV40-induced antigens (Fig. 2): tumoral antigens (or T antigen) for $TSV_5Cl_2$, ZDL, $CT_{54}$ and membrane antigens.

The spleen sections of TBH where shown (de Vaux St Cyr *et al.*, 1977) to contain numerous IgG-synthesizing cells; at the beginning of the tumour graft they are plasma cells; at the end, they are young lymphocytic cells.

FIG. 1.—Spleen section (1·5 μm). A solitary tumour cell is seen. ×400.

FIG. 2.—Spleen section (1·5 $\mu$m). A tumour cell is stained by an antiserum against SV40-induced antigen $\times 400$.

The spleens of hamsters bearing tumours of different origin (EHB, $TSV_5Cl_2$, ZDL, $CT_{54}$, Na) consistently contain neoplastic cells scattered throughout the splenic reticulum from early in the growth of the tumour. Their number increases in proportion to the weight of the tumour. The presence of tumour cells at the centre of an organ housing cells of immunological function poses several problems: are they alive, and why?

When examined either histologically or by fluorescence, tumour cells are rarely seen in the process of destruction. Furthermore, the grafting or the culture of these splenic tissues, even in the case of tumours of minimal weight, allows the multiplication of cells resembling those of the original tumour. They are therefore malignant and living.

The presence of thymus-dependent cells in the periarteriolar spaces at the outset of the growth of the tumour graft is not, however, sufficient in itself to destroy all the tumour cells in the spleen. We must state (unpublished results) that in this tumour system it has never been possible to demonstrate *in vitro* cell-mediated cytotoxicity (CMC) either by $^{51}Cr$ (Brunner *et al.*, 1976) or $^{3}H$-proline release (Saal *et al.*, 1976).

For reasons at present unknown, it seems that the CMC functions poorly in the spleen in the early stages of the splenic evolution of a tumour bearer. This may be more understandable in the late stage when the thymus-dependent areas have disappeared (de Vaux St Cyr *et al.*, 1977).

Another question is: Why do the cells remain isolated?

The presence of a cytostatic factor might explain why the tumour cells are not organized into metastatic foci in the spleen. Possibly the cells are maintained in $G_0$ either by cells with cytostatic activity or by a secreted factor. Their culture or grafting may allow them to recover their proliferative capacity.

As cytostatic factors one could hypothesize suppressive cells, humoral antibodies or antigen–antibody complexes, the presence of which has been demonstrated particularly in the case of tumours induced by the inoculation of $TSV_5Cl_2$ cells.

But the best candidates are the cells with cytostatic activity on tumour cells, which have been demonstrated by Haddada *et al.* (in preparation).

It may be also asked whether the spleen of TBH is a reservoir for malignant cells.

After tumour resection, considering the early presence of tumour cells in the spleen, metastasis might develop by release of these tumour cells into the bloodstream. This might pose an important medical problem if a similar phenomenon also occurred in man.

### REFERENCES

BRUNNER, K. T., ENGERS, H. D. & CEROTTINI, J. C. (1976) The $^{51}Cr$ release assay as used for the quantitative measurement of cell mediated cytolysis *in vitro*. In: In Vitro *Methods in Cell Mediated and Tumour Immunity*. Ed. Bloom, B. R., and David, J. R. New York: Academic Press. p. 423.

HELLSTRÖM, I. & SJÖGREN, H. O. (1965) Demonstration of H-2 antigens and polyoma specific tumor antigens by measuring colony formation *in vitro*. *Exp. Cell Res.*, **40**, 212.

LOISILLIER F., ZUINGHEDAU, J. & DE VAUX ST CYR, C. (1977) An anatomopathological study of the lymphoid system in hamsters during the growth of an SV40-induced tumour. *Br. J. Exp. Path.*, **58**, 533.

MEYER, G., LHERISSON-STABONI, A. M. & BONNEAU, H. (1969) Sensibilité différentielle aux rayons ultraviolets de quelques fonctions du virus polyome. *Int. J. Cancer*, **4**, 520.

SAAL, J. G., RIEBER, E. P. & REITH MÜLLER, G. (1976) Cell-mediated cytotoxicity for melanoma tumor cells. Detection by a $^{3}H$-proline release assay. *Scand. J. Immunol.*, **5**, 455.

TOURNIER, P., CASSINGENA, R., WICKER, R., COPPEY, J. & SUAREZ, H. (1967) Etude du mécanisme de l'induction chez des cellules de hamster syrien transformées par le Virus SV40. Propriétés d'une lignée cellulaire clonale. *Int. J. Cancer*, **2**, 117.

DE VAUX ST CYR, C., LOISILLIER, F. & ZUINGHEDAU, J. (1977) Humoral and cellular immune response during the growth of an SV40 induced tumour in hamsters. *Ann. Microbiol. (Paris)*, **128B**, 385.